# Reviewed article: Siqueira PFS, Sousa DBP, Moreira AFG, Marchi BS, Teixeira GC, Martins PF, Alves WA. Deaths from infectious respiratory diseases in the Indigenous population: spatial and time series analysis, Minas Gerais, 2000-2023. Epidemiol Serv Saude. 2026:35;e20250565

**DOI:** 10.1590/S2237-96222026v35e20250565.a

**Published:** 2026-04-20

**Authors:** Lucas Vinícius de Lima

**Affiliations:** 1 Secretaria Municipal da Saúde, Gerência de Planejamento, Maringá, PR, Brazil Secretaria Municipal da Saúde Gerência de Planejamento Maringá PR Brazil

## Peer review A 

## Questionnaire 

Does the manuscript contain new and significant information to justify publication? Yes 

Does the Abstract (Summary) clearly and accurately describe the content of the article? Yes 

Is the problem significant and concisely stated? Yes 

Are the methods described comprehensively? No 

Are the interpretations and conclusions justified by the results? Yes 

Is adequate reference made to other work in the field? Yes 

## Manuscript Structure 

Length of article is: Adequate 

Number of tables is: Adequate 

Number of figures is: Adequate 

Please state any conflict(s) of interest that you have in relation to the review of this paper. None 

Interest: Good 

Quality: Average 

Originality: Good 

Overall: Good 

Recommendation: Major Revision 

## Comments 

Trata-se de um estudo de base ecológica com recorte étnico-racial relevante e abordagem temporal e espacial pertinente, voltado à análise dos óbitos por doenças respiratórias infecciosas entre a população indígena em Minas Gerais. O manuscrito é oportuno, especialmente frente às lacunas de informação sobre a saúde indígena no Brasil, e apresenta potencial para contribuir com a vigilância em saúde e o planejamento de ações direcionadas a populações vulnerabilizadas. As sugestões a seguir visam qualificar ainda mais o trabalho, mantendo seu mérito temático e ampliando sua robustez científica. 

Título: 

- Considerando o comentário nos métodos de que o estudo não analisa efetivamente a mortalidade, que implica, por definição, o uso de uma taxa, sugiro readequar o título para: “Tendência temporal e distribuição espacial dos óbitos por doenças respiratórias infecciosas na população indígena em Minas Gerais: análise espacial e de séries temporais, 2000-2023”. 

Introdução: 

- No início da seção, quando os autores contextualizam a problemática das infecções respiratórias, seria oportuno trazer um conceito geral que caracterize esse grupo de doenças, seja pela manifestação clínica, seja pela forma de transmissão. Isso ajudaria o leitor a ter uma familiaridade maior com o entendimento dos autores no que tange às doenças. 

- Embora os autores citem o Fórum Internacional de Sociedades Respiratórias, faltam dados quantitativos globais e nacionais sobre a carga dessas doenças (mortalidade, internações, etc.), como forma de demonstrar a magnitude do problema. Sugiro incluir dados atualizados da Organização Mundial da Saúde (OMS) ou do Global Burden of Disease Study para fortalecer. 

- Os autores mencionam a relação entre a população indígena e as desigualdades em saúde, mas faltam referências mais explícitas sobre as barreiras estruturais enfrentadas, como: acesso geográfico, precariedade de unidades básicas de saúde em áreas indígenas, carência de profissionais e medicamentos, desassistência em vigilância e diagnóstico, etc. 

- No momento em que os autores falam a respeito da aglomeração intradomiciliar, trazendo à tona a quantidade de moradores/habitantes por residência, seria interessante apresentar os dados nacionais e estaduais de densidade domiciliar para o contexto da população geral, como forma de comparar e dar ênfase no valor da população originária. 

- Quando os autores abordam o cenário da covid-19 e da síndrome respiratória aguda grave (SRAG), seria oportuno trazer dados epidemiológicos do cenário da infecção na população geral e indígena, no país e no estado. Essa complementação ajudaria no argumento que os autores tentaram introduzir, no que se referente ao acometimento mais intenso. 

- A justificativa menciona a “escassez de investigações”, mas não explicita o que diferencia este estudo dos demais nem qual a principal lacuna que ele se propõe a preencher. Uma busca em sites de busca retorna vários artigos (https://doi.org/10.1590/1413-812320242912.09392024; https://doi.org/10.1590/S0104-12902022210368pt; https://doi.org/10.1590/S2237-96222025v34e20240176.pt, etc.), então seria importante delimitar o seu diferencial. 

- A introdução transita entre dados demográficos, pandemia, situação sanitária e a estrutura do Sistema Único de Saúde (SUS), mas essas ideias ainda estão pouco articuladas. Nesse sentido, sugiro incluir frases de conexão ou síntese ao final dos parágrafos para orientar o leitor na progressão lógica da argumentação em defesa pelos autores. 

Métodos: 

- Os autores mencionam que realizaram um “estudo de análise de série temporal”. Sugiro que seja reformulado para “estudo de séries temporais e múltiplos grupos”, visto que foram realizadas análises em função do tempo e do espaço. Segue uma sugestão leitura a respeito das tipologias de estudo ecológico: https://doi.org/10.1146/annurev.pu.16.050195.000425. 

- Sugiro revisar o uso do termo “mortalidade” ao longo do manuscrito. Considerando que não foram utilizadas estimativas populacionais da população indígena sob risco, o termo pode ser tecnicamente inadequado. Expressões como “óbitos registrados” e “número de óbitos” refletem de forma mais precisa a natureza descritiva dos dados analisados, evitando interpretações equivocadas sobre o risco de morte. 

- Sugiro que os autores revisem as macrorregiões de saúde mencionadas no subitem “local do estudo”. Na minha contagem, foram apresentadas quinze regiões, e não dezesseis, conforme o informado. Analisando a Deliberação CIB/MG n.º 4.394/2023, parece que a região faltante foi a Centro Sul (sugiro revisar o link de acesso na referência pois não funcionou). 

- O estudo considerou apenas a causa básica de morte, conforme codificada na declaração de óbito (DO). No entanto, é possível que doenças respiratórias infecciosas tenham sido registradas como causas associadas ou contribuintes, especialmente em casos com múltiplas comorbidades ou em que a causa básica foi registrada como “insuficiência respiratória”, “sepse”, ou outra complicação genérica. Sugiro pontuar como uma limitação. 

- De acordo com a iniciativa Strengthening the Reporting of Observational Studies in Epidemiology (STROBE), para cada variável do estudo, deve ser fornecida as categorias de respostas. Por exemplo, ao apresentar as variáveis de caracterização (sexo, faixa etária, estado civil, etc.), seria importante complementar: sexo (masculino; feminino), etc. 

- Embora os autores indiquem que os critérios de inclusão visaram reduzir viés, é importante observar que a exclusão de registros com causas associadas, etiologia inconclusiva ou raça/cor ignorada pode introduzir viés de seleção e de informação, especialmente em populações com menor acesso ao diagnóstico ou ao adequado preenchimento da DO. Recomendo reavaliar esse trecho e reconhecer essa limitação de forma mais explícita na discussão. 

- É importante refletir sobre a suposição de homoscedasticidade. Trata-se de uma série temporal de óbitos, na qual é comum haver autocorrelação serial entre os anos consecutivos. A ausência de uma verificação explícita da presença de autocorrelação serial nos resíduos pode comprometer a validade dos intervalos de confiança e dos testes de significância, superestimando os erros tipo I (https://doi.org/10.5123/S1679-49742015000300024). 

- A escolha de utilizar o número absoluto de óbitos como variável dependente na regressão joinpoint, embora operacionalmente mais simples, pode comprometer a interpretação dos resultados, uma vez que não considera variações no tamanho da população indígena ao longo dos anos e entre regiões. Recomendo que os autores justifiquem essa escolha metodológica, especialmente considerando que a literatura recomenda o uso de taxas de mortalidade. 

- Recomenda-se explicitar o critério estatístico usado para a escolha do modelo na regressão joinpoint (como critério de informação bayesiano ponderado ou teste de permutação) na definição do número de segmentos (joinpoints) em cada série histórica analisada, a fim de garantir transparência e reprodutibilidade da análise. 

- A imputação de valores artificiais (0,10) nos anos com ausência de óbitos pode ser metodologicamente questionável. Tal abordagem pode distorcer as estimativas de tendência e amplificar as variações em séries com baixa frequência de eventos. Sugiro que os autores considerem, como alternativa, o uso de média móvel de três pontos, técnica comumente adotada para suavizar flutuações aleatórias em séries de dados raros. 

- Recomendo disponibilizar como material suplementar a lista de códigos da Classificação Internacional de Doenças (10ª edição) utilizados para classificar o agente etiológico e o sítio anatômico da infecção, com indicação de como cada código foi alocado. Isso ajudaria na transparência da categorização adotada, facilitando o entendimento. 

- A divisão espacial por três grandes períodos (2000-2009, 2010-2019, 2020-2023) carece de justificativa teórica ou empírica. Por que esses cortes e não outro (ex.: considerando inflexões reais observadas na série)? Nesse sentido, como as análises já foram realizadas, sugiro justificar os critérios usados para definir os períodos da análise espacial (três blocos). 

- Embora o uso das macrorregiões de saúde seja coerente com o planejamento estadual, seria interessante discutir a limitação do uso dessas divisões administrativas para representar a distribuição real da população indígena, que se organiza territorialmente a partir de terras indígenas e distritos sanitários especiais indígenas. Seria interessante sinalizar nos mapas as regiões em que há esses agrupamentos, por meio de algum centroide. 

- Os autores mencionam o uso de “teste de proporções” nas análises de comparação das características. Recomendo especificar o nome exato do teste estatístico utilizado (ex.: teste Z, teste qui-quadrado de Pearson ou teste exato de Fisher, conforme os pressupostos), a fim de garantir clareza metodológica e reprodutibilidade do estudo. 

Resultados: 

- Embora a seção de resultados esteja bem detalhada e apresente adequadamente os dados obtidos, observa-se que há um excesso de dados numéricos descritos de forma textual, o que torna a leitura por vezes densa e exaustiva. Considerando que os valores absolutos e percentuais já estão organizados de maneira clara nas tabelas, recomenda-se que a descrição no texto seja mais sintética, destacando apenas os achados mais relevantes e significativos. 

- O modelo joinpoint gerou resultados detalhados, mas falta uma síntese geral das tendências predominantes e regiões com padrões divergentes. Sugiro acrescentar uma frase-resumo para referenciar a tabela com as tendências, destacando quais regiões apresentaram aumento, redução ou estabilidade - e em que períodos, com foco nos padrões e nas divergências. 

- Caso seja mantida, a imputação de 0,10 óbito em anos sem registros, embora metodologicamente citada, deve ser interpretada com cautela nos resultados, para que o leitor não os entenda como eventos reais. Recomendo sinalizar de forma clara como nota de rodapé da tabela quais foram as séries históricas que precisaram ter valores imputados. 

- Nas tabelas em que são empregados os testes de hipótese para comparação das frequências, sugiro que os autores indiquem/sinalizem, por meio de símbolo sobrescrito/letra sobrescrita qual foi o teste aplicado em cada situação, a fim de garantir clareza metodológica e reprodutibilidade do estudo (similar ao feito na Tabela 2). 

- A Tabela 3 está um pouco confusa. A coluna “classificação da tendência”, ao se referir à variação anual, deveria estar imediatamente após ela. Uma opção seria colocar apenas como nota: “Valores positivos de variação percentual, desde que estatisticamente diferentes de zero (considerando o intervalo de confiança), indicam tendência crescente; valores negativos indicam tendência decrescente. Os demais foram interpretados como tendência estacionária”. 

- Recomendo rever a apresentação conjunta de p-valores e intervalos de confiança nas estimativas de tendência, uma vez que ambos expressam a significância estatística da mesma forma. Quando o intervalo de confiança é apresentado (preferível, inclusive), o p-valor pode ser dispensado, evitando redundância na exposição dos resultados e contribuindo para uma apresentação mais concisa, interpretativa (magnitude e significância) e objetiva. 

- Nas Figuras 1 e 2, além da sugestão de inserção dos centroides indicativos das terras indígenas ou dos distritos sanitários especiais indígenas, recomenda-se a sobreposição de uma nova camada geográfica delimitando as dezesseis macrorregiões de saúde. Essa demarcação contribuiria para alinhar visualmente os dados espaciais às análises temporais, que foram estratificadas por macrorregião, favorecendo a coerência entre os diferentes eixos analíticos. 

Discussão: 

- Recomendo que a seção de discussão seja iniciada com um parágrafo-síntese dos principais achados do estudo, conforme as orientações da iniciativa STROBE. Esse parágrafo deve retomar de forma concisa os resultados mais relevantes da análise temporal e espacial da mortalidade, sem repeti-los em detalhes/valores, servindo como base para a interpretação e a discussão aprofundada nos parágrafos subsequentes. 

- O texto menciona referências, mas não explora contrastes ou convergências relevantes com a literatura. Sugiro discutir se os padrões identificados (ex.: maior mortalidade na região “x”, predominância de causas não especificadas, etc.) são similares ou divergentes dos encontrados em outros estados, em populações indígenas não mineiras, ou na população geral. 

- Algumas macrorregiões apresentaram tendências estacionárias ou oscilações nos indicadores sem um padrão claro ou consistente ao longo do tempo. Recomendo que os autores explorem possíveis explicações para esses comportamentos, como a ocorrência de subnotificações ou falhas no preenchimento da DO, a eventual migração de indivíduos indígenas entre municípios ou estados, ou ainda diferenças na cobertura e acesso aos serviços de saúde. 

- A discussão toca em vulnerabilidades sociais e em estratégias de proteção social, mas de modo superficial. Sugiro explorar mais o papel da precariedade estrutural (saneamento, água, barreiras geográficas e linguísticas, preconceito, etc.) e como isso pode se traduzir em maior mortalidade, bem como discutir as estratégias governamentais para atenuar esses contextos. 

- O texto menciona a necessidade de políticas públicas, mas fica um pouco vago. Pode-se indicar o que os achados implicam para a vigilância epidemiológica, para a atuação da Secretaria Especial de Saúde Indígena, para o fortalecimento da atenção primária nas áreas indígenas, ou mesmo para qualificação da DO. 

- A discussão aborda limitações (ex.: dados secundários), mas poderia ser mais autocrítica. Recomendo apontar que a análise se restringiu à causa básica, não utilizou taxas padronizadas por falta de dados populacionais indígenas por município e que a heterogeneidade dos dados pode afetar a robustez das tendências, além de outras reflexões pontuadas nos métodos. 

- Dado o recorte étnico-racial adotado, sugiro que a discussão inclua uma reflexão sobre a importância da qualificação dos sistemas de informação para a população indígena, especialmente no que se refere ao preenchimento da variável raça/cor e à inclusão de marcadores territoriais mais sensíveis à organização sociocultural indígena. 

- Recomendo finalizar a discussão indicando que os achados podem subsidiar estudos futuros mais aprofundados sobre mortalidade indígena, de forma mais enfática e propositiva, bem como ações específicas de vigilância e planejamento em saúde voltadas à proteção dessa população, com base em evidências georreferenciadas e culturais.

